# Be Well Communities™: mobilizing communities to promote wellness and stop cancer before it starts

**DOI:** 10.1007/s10552-021-01439-9

**Published:** 2021-05-26

**Authors:** Ruth Rechis, Katherine B. Oestman, Elizabeth Caballero, Anna Brewster, Michael T. Walsh, Karen Basen-Engquist, Jeffrey E. Gershenwald, Jennifer H. Tektiridis, Mark Moreno, Pamela A. Williams, Katherine Treiman, Priscila D. Garza, Ernest Hawk

**Affiliations:** 1 Cancer Prevention and Control Platform, Unit 1364, The University of Texas MD Anderson Cancer Center, 1515 Holcombe Boulevard, Houston, TX 77030 USA; 2 MD Anderson Physicians Network, Houston, TX USA; 3 Division of Cancer Prevention & Population Sciences, Houston, TX USA; 4 Department of Surgical Oncology, Houston, TX USA; 5 Governmental Relations, Houston, TX USA; 6 RTI International, Research Triangle Park, NC USA; 7 Goose Creek Consolidated Independent School District, Baytown, TX USA

**Keywords:** Population health, Risk reduction behavior, Cancer prevention, Implementation science, Community outreach and engagement

## Abstract

**Purpose:**

Increasingly, cancer centers are delivering population-based approaches to narrow the gap between known cancer prevention strategies and their effective implementation. Leveraging successful healthy community initiatives, MD Anderson developed Be Well Communities™, a model that implements evidence-based actions to directly impact people’s lives.

**Methods:**

In partnership with local organizations, MD Anderson’s Be Well Communities team executed and evaluated 16 evidence-based interventions to address community priorities in healthy diets, physical activity, and sun safety. Evaluation included assessing the effectiveness of evidence-based interventions, stakeholders’ perceptions of collaboration, and the population-level impact on dietary and physical activity behaviors among students using the School Physical Activity and Nutrition Survey and the System for Observing Fitness Instruction Time. Two-tailed *t*-tests were used to compare tested parameters at baseline and follow-up. *p* values less than .05 were considered significant.

**Results:**

This model achieved its early outcomes, including effectively implementing evidence-based interventions, building strong partnerships, increasing access to healthy foods, improving the built environment, and increasing healthy food and water consumption and moderate to vigorous physical activity among students (*p* < .001).

**Conclusions:**

Be Well Communities is an effective model for positively impacting community health which could be leveraged by others to deliver evidence-based actions to improve population health.

## Introduction

The USA has a rich history of coalitions improving the health of communities [1]. Collaborations with engagement in communities that are designed to impact population health are often referenced as healthy community initiatives [2–4]. Two leading healthy community initiatives are Shape Up Somerville [5] and the Centers for Disease Control and Prevention’s Healthy Communities Program [6]. Although engaging communities in cancer prevention could reduce the cancer burden, few healthy community initiatives in the USA have focused specifically on cancer prevention or been led by a comprehensive cancer center. One important exception is the SF Can [7].

Over the past several decades, important advances have been made in cancer prevention. Recent research estimates that as much as 50% of cancer cases could be preventable by more consistently applying current knowledge to the population [8], yet a tremendous gap exists between this knowledge and implementation of effective prevention strategies for at-risk populations. Modifying lifestyle behaviors can reduce cancer risk and may reduce the cancer burden by 40% [9]. Beyond primary prevention, additional lives can be saved by implementing evidence-based screening to diagnose cancer at its earliest and most curable stage [10–12]. However, these screening strategies typically require diagnostic evaluations and surgical interventions following early detection, and are not consistently practiced, especially in low-resource communities. A critical step in reducing cancer incidence is to effectively employ evidence-based interventions for prevention at the population level.

Cancer centers are uniquely positioned to improve the public’s health by conducting relevant research with communities, concentrating on discovery and implementation of scientific findings, and serving as champions to fuel consistent broad community adoption of evidence-based actions [13]. Increasingly, cancer centers, hospitals, and other providers are being asked to integrate population-based approaches into their operations [14]. Influential organizations have issued calls to implement evidence-based interventions to improve the nation’s health. One example was the Surgeon General’s Call to Action to Prevent Skin Cancer [15], which outlined strategies for individuals, institutions, and communities to reduce ultraviolet radiation (UVR) exposure.

Legislatures and accreditors issued mandates to healthcare institutions for community engagement to better align medical care and public health [16]. The US Patient Protection and Affordable Care Act includes community benefit requirements for non-profit hospitals [17], and the Commission on Cancer [18] includes requirements for community assessment and engagement. Furthermore, in 2016, the National Cancer Institute made community outreach and engagement a critical criterion for National Cancer Institute-designated cancer centers [19]. Although these centers have traditionally focused on research, patient care, and education, this mandate recognizes that they are well positioned to improve population health and requires them to demonstrate how they engage within their catchment areas. Herein, we outline one approach by which The University of Texas MD Anderson Cancer Center is addressing community outreach and engagement through a healthy community initiative.

MD Anderson has been committed to cancer prevention for more than 40 years, a commitment recognized as vital to its mission to end cancer. In 2012, MD Anderson launched the Moon Shots Program, an effort to accelerate the translation of scientific discoveries into clinical and public health advances that save lives. This program established the Cancer Prevention and Control Platform, which implements evidence-based interventions involving community services, public education and policy interventions, targeting measurable reductions in cancer incidence and mortality. This platform initiated Be Well Communities™ to mobilize communities to promote wellness and address modifiable cancer risk factors. Specifically, Be Well Communities focuses on five target areas aligned with cancer risk reduction: tobacco use, physical inactivity, unhealthy diets, UVR exposure, and inadequate preventive care (e.g., cancer screening, human papillomavirus vaccination) [9].

In 2014, MD Anderson received a gift from ExxonMobil to support implementation of a healthy community model focused on modifiable cancer risk factors in Harris County, Texas. The inaugural Be Well Community was Baytown (Be Well™ Baytown), which is located 30 miles east of Houston. Table 1 includes demographics for Baytown, a medically underserved community with relatively high rates of unhealthy behaviors, as compared to Healthy People 2020 goals. Selection of the location was based on identifying a community with needs associated with elevated cancer risks (e.g., high rates of obesity) balanced with the capacity to address those needs. Be Well Baytown, began in 2017 and will be active through 2025. The first 2 years and early results of its implementation are discussed below.

**Table 1:** Baytown demographics and health indicators related to cancer risk compared with Healthy People 2020 goals

Total population*	%	
Age		
< 5 years	7.7	
5–17 years	21.1	
18–64 years	59.4	
≥ 65 years	11.8	
Race		
White	32.3	
Black	18.6	
Hispanic	44.6	
Sex		
Male	50.4	
Female	49.6	

Health indicator (age range)	Baytown (%)	Healthy People 2020 goal (%)^a^
Mammography use among women (50–74 years)	75.4^b^	81.1
Papanicolaou smear use among women (21–65 years)	80.9^b^	93.0
Up-to-date colorectal cancer screening among adults (50–75 years)	57.7^b^	70.5
Up-to-date human papillomavirus vaccination among adolescents (13–17 years)	55.2^c^	80.0
Obesity among adults (18 + years)	34.4^b^	30.5
Obesity among children (12–17 years)	22.0^d^	14.5
Current smoking among adults (18 + years)	18.9^b^	12.0
No leisure-time physical activity among adults (18 + years)	32.1^b^	32.6

*US Census Bureau: American Community Survey, 2018

^a^US Department of Health and Human Services: Healthy People 2020

^b^500 Cities: Local Data for Better Health, 2016–2017

^c^National Immunization Survey, 2017

^d^Health of Houston Survey, 2010

## Methods

### Establishing the infrastructure

The Be Well Communities model builds on lessons learned from successful healthy community initiatives and from the Collective Impact model, which demonstrated that more can be achieved when organizations work collaboratively toward a common goal [20]. One key finding of this prior work was the need to establish a specific vision and infrastructure to support implementation, built on the community’s strengths which could lead to the community managing and sustaining the initiative over time [21].

**Fig. 1:**
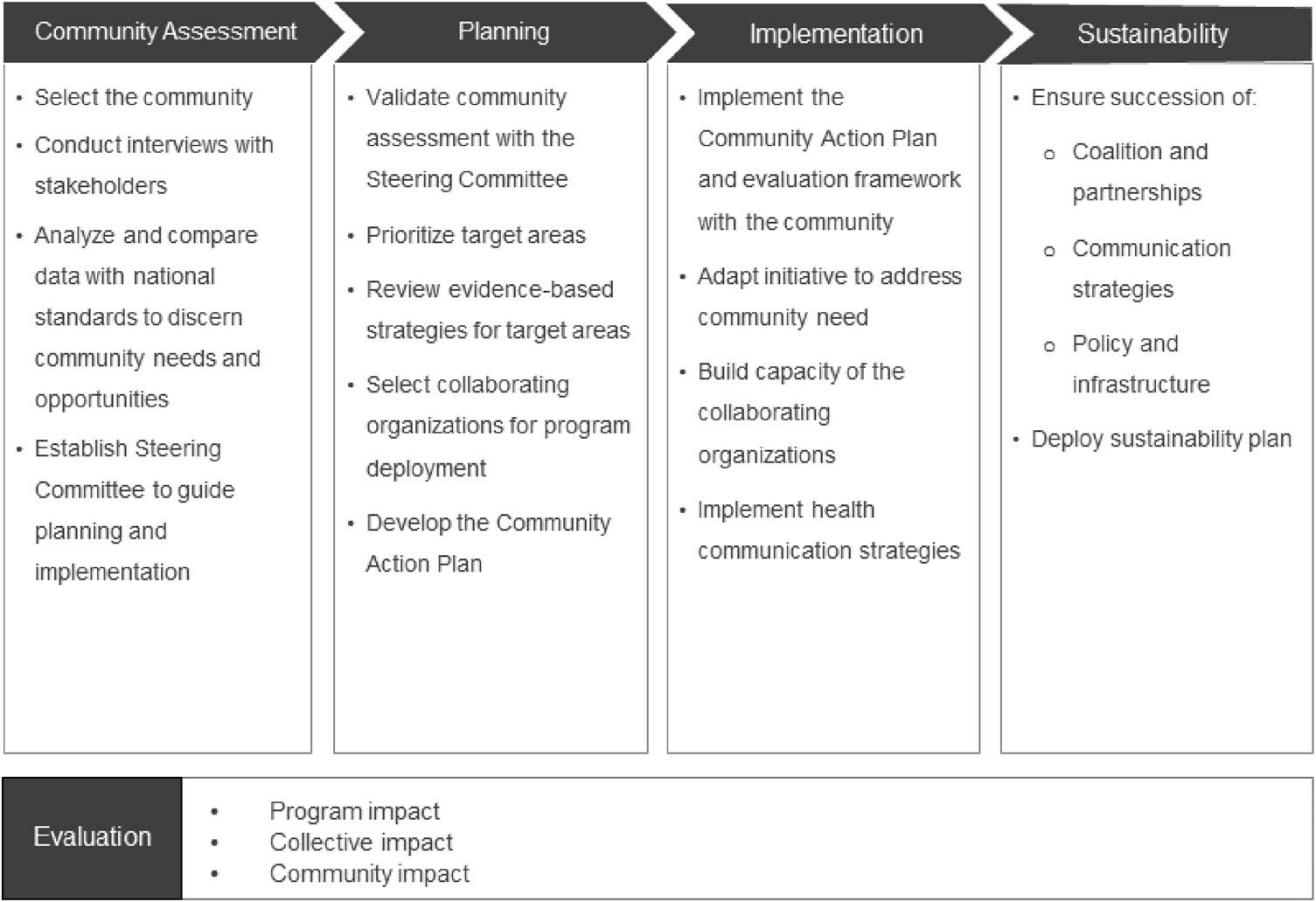
Stepwise process and overall components of the Be Well Communities model

The Be Well Communities model engages communities throughout the process and ultimately empowers them to lead and sustain the work. Figure 1 outlines the model which includes the initial community assessment stage, a planning stage, and an implementation stage. Sustainability is considered from the outset and evaluation is included at all stages.

The model relies on three groups working collaboratively: a backbone team, a Steering Committee, and collaborating organizations.

In Baytown, MD Anderson’s Be Well Communities team served as the backbone team to foster cross-sector communication, alignment, and collaboration. The backbone team was responsible for the following:

- Envision and catalyze a coordinated, collaborative approach to health with the community
- Conduct the foundational community assessment
- Provide dedicated staff to support program implementation, participate actively in the community, identify additional funding opportunities, coordinate across organizations/coalitions, and steward the Steering Committee and the working groups
- Provide funding to collaborating organizations to strengthen existing or support new programs, implement infrastructure changes, and/or support staff to implement programs
- Provide ongoing training, technical assistance, and capacity-building to support the execution of evidence-based interventions
- Create, execute and evaluate a sustainable Community Action Plan (CAP) in partnership with the community that can be carried out by the community over the long term
- Work with all organizations to develop, adapt, execute and evaluate the CAP to ensure it is addressing the needs of the community
- Deliver communication strategies to share information and connect people with resources
- Provide access to healthcare and wellness resources
- Create a sustainability plan to transition the initiative to the community
- Meet with partners to identify, solve issues often by connecting to additional resources, and to identify opportunities for organizations to work together
- Review and approve quarterly reports from the collaborating organizations
- Maintain and share a database of evidence-based interventions for program deployment, emphasizing the importance of evidence-based interventions
- Connect with MD Anderson faculty and staff for strategic scientific expertise and guidance
- Leverage internal MD Anderson resources to support legal agreements, health communication strategies and best practices

The Steering Committee includes community champions from non-profits, businesses, schools, healthcare institutions, city officials, and residents. The Steering Committee was responsible for the following:

- Attend monthly meetings
- Connect the initiative to the community
- Provide strategic guidance
- Monitor and support program implementation
- Review and prioritize new program implementation
- Champion Be Well Baytown in the community
- Participate in annual Stakeholder surveys

The Be Well Baytown Steering Committee was the first ever health coalition in this community composed of 25 individuals from 16 organizations. While individual organizations conducted health-related activities prior to the start of this work, those prior efforts were neither coordinated, nor led by a collaborative group focused on improving health of the overall community as a shared goal. By working together to select projects, support each other, hold each other accountable, and align on mutually reinforcing actions, the collective actions of this committee support the delivery of evidence-based interventions.

Collaborating organizations are Steering Committee organizations that submit proposals and are competitively selected for funding to implement and evaluate evidence-based interventions. The six collaborating organizations were responsible for the following:

- Deliver evidence-based interventions in the community
- Provide monthly updates to the Steering Committee
- Work collaboratively with members of the Steering Committee
- Submit quarterly progress reports to the backbone team
- Participate in interviews or other evaluation strategies
- Develop a strategy to sustain programs after funding ends

### Prioritizing target areas

**Fig. 2:**
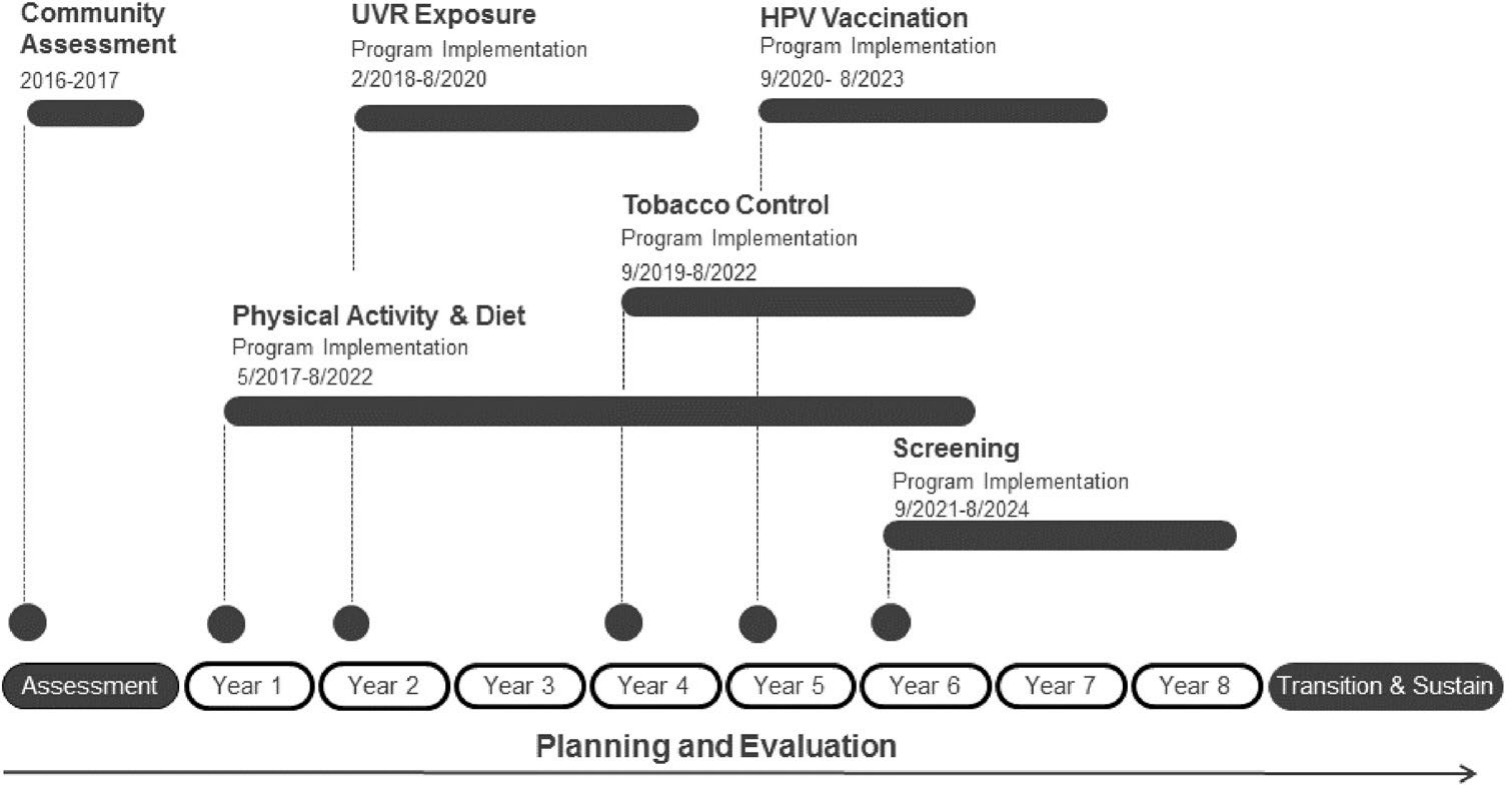
Timeline of the community assessment process, program implementation, and sustainability for Be Well Baytown

At the first Steering Committee meeting, attendees reviewed and validated information that had been gathered about the community including demographics, cancer relevant behavioral/health data, and available resources (e.g., parks, schools). Next, the Steering Committee prioritized which of the five target areas to address first based on the information that was gathered about the community, their perceptions of community need, and the current status of implementation regarding evidence-based interventions. The Steering Committee prioritized healthy eating and physical activity because it was an area that could impact all residents. When reviewing the community assessment data with the Steering Committee, the discrepancy between the childhood and adult obesity rates for Baytown versus the Healthy People 2020 goals was identified as an area of concern that should be the initial focus for this project. Critical to the success of this initiative is the guidance from residents themselves to ultimately promote a culture of health that is led and sustained by and for the community. Figure 2 shows the timeline for addressing each target area over the next several years. While implementation of dietary, physical activity, and sun safety measures continue; at the writing of this article, the implementation of additional programs involving tobacco control and HPV vaccination are underway.

### Community action plan

After selecting the target areas, the Steering Committee reviewed evidence-based interventions and agreed upon which interventions would be most appropriate for the community. To guide this discussion, the backbone team provided its database of evidence-based interventions which includes resources from the Robert Wood Johnson Foundation [22], the Centers for Disease Control and Prevention’s Guide to Community Preventive Services [23], Evidence-based Cancer Control Programs (Formerly RTIPs) website which houses the evidence-based interventions on Cancer Control P.L.A.N.E.T. [24], and critical reports, such as those from the Institute of Medicine [25].

Next, Steering Committee organizations had the opportunity to submit a proposal for funding to become a collaborating organization that implements and sustains one or more evidence-based interventions to advance the community’s health. Proposals were competitively vetted to assess alignment as evidence-based interventions, sustainability, and fiscal management by MD Anderson and external experts in cancer prevention and community engagement. In their role of providing strategic guidance, proposals were also reviewed by the Steering Committee, excluding their own submissions, to assess potential interest from the community and to ensure they represented new or enhanced programmatic activities that did not duplicate existing funded activities currently in the community.

Together, the approved proposals created the CAP for Be Well Baytown outlining the specific work to be carried out from 2017 to 2019. Table 2 provides a high-level overview of the CAP, the types and number of evidence-based interventions, activities, and intended outcomes. Six collaborating organizations were selected to execute 16 evidence-based interventions for diet, physical activity, and UVR exposure.

**Table 2:** High-level overview of the community action plan (CAP) for Be Well Baytown—evidence-based interventions, activities, program impact, and long-term health-related outcomes

Diet and physical activity			
Collaborating Organizations: United Way of Greater Baytown Area and Chambers County, Hearts and Hands of Baytown Food Pantry, GCCISD, YMCA, HCPH Total Number of Evidence-Based Interventions: 11			
Evidence-Based intervention [22, 23]	Activity	Program impact	Anticipated long- term health-related outcomes
Healthy food initiatives in food banks Multi-component school-based obesity prevention interventions School-based PE School-based nutrition education programs Physically active classrooms Screen time interventions for children Extracurricular activities for physical activity Nutrition and physical activity interventions in preschool and child care settings Places for PA Community fitness programs Community-based social support for PA	Deliver 300,000 lb of produce through remote healthy food initiatives annually Hire a Healthy Schools Coordinator for GCCISD Implement CATCH in all elementary and junior high schools Deliver YMCA after-school programs Enhance indoor and outdoor learning environments (OLE! Texas) at 3 early child-care centers Build 4 walking trails for the community Implement 5 YMCA walking clubs Implement YMCA’s Diabetes Prevention Program	Over 1 million pounds of fresh produce delivered to approximately 42,000 families 12,103 GCCISD students participated in CATCH 338 youth participated in YMCA after-school sports 3 early child-care facilities selected for Outdoor Learning Environments (OLE)! 4 community walking trails were built 155 participants in the YMCA’s walking clubs 61 participants in the YMCA’s Diabetes Prevention Program	Increased Healthy food consumption Access to healthy food Increased PA Access to places for PA

UVR exposure			
Collaborating Organizations: COB, GCCISD, Lee College, HCPH Total Number of Evidence-Based Interventions: 5			
Evidence-Based Intervention [23]	Activity	Program Impact	Anticipated Long-Term Health-related Outcomes
Multicomponent community-wide interventions to prevent skin cancer Interventions in outdoor occupational settings Interventions in outdoor recreational and tourism settings Child care center-based interventions Primary and middle school-based interventions	Deliver classes on sun safety for outdoor workers Provide wide-brimmed hats for COB outdoor workers to reduce sun exposure Install 10 sunscreen dispensers and 5 shade structures and increase natural shade in parks Execute a school district-wide sun safety policy Deliver school-based sun safety lessons Install 10 sunscreen dispensers at Lee College Deliver sun safety classes and social media messages and promote UV protection across the community in partnership with Lee College, COB, and HCPH	95 outdoor workers were trained on sun safety 100 hats were provided 20 sunscreen dispensers and 6 shade structures were installed at schools and in parks 3,318 GCCISD students received sun safety education and a sun safety policy was developed and implemented 7,500 Lee College students and staff were provided access to sunscreen and sun safety programming 83% of the community received messages about sun safety	Increased Knowledge of sun-protective behaviors Use of sunscreen and protective clothing Access to environmental or policy approaches to encourage sun protection Decreased UVR exposure

*HCPH* Harris County Public Health, *PA* physical activity, *COB* City of Baytown, *GCCISD* Goose Creek Consolidated Independent School District

The Steering Committee’s strategic guidance prioritized efforts based in public schools because of their exceptional reach within the community. This is consistent with past healthy community initiatives and is rooted in the Association of Supervisors and Curriculum Development and the Centers for Disease Control and Prevention’s Whole School, Whole Community, Whole Child model [26]. This model focuses on the child, but acknowledges the critical role that community agencies and families play in improving childhood health behaviors and development. Systematic reviews demonstrated that diet and physical activity interventions based in a school component are effective at preventing obesity [27]. From a cancer prevention focus, early intervention is critical, as cancer risk in adults increases in relation to childhood body mass index [28]. Similarly, childhood sunburns are a significant risk factor for skin cancer later in life [29]. Maintaining a healthy weight, being physically active, eating a healthy diet, and protecting skin against UVR overexposure early are indispensable cancer prevention activities that can be adopted during childhood and effectively implemented in schools [15, 30].

One program known to be effective in addressing childhood obesity and its downstream impact is the Coordinated Approach to Child Health (CATCH®) program. Substantial evidence supports the effectiveness of CATCH in increasing physical activity and healthy eating [31, 32] and reducing excessive weight and obesity [33–35] by enhancing the messaging a child receives in physical education (PE) classes, the lunchroom, the classroom, and at home. CATCH PE aims to increase moderate to vigorous physical activity (MVPA) during PE classes. Consistent with the recommended national guidelines, the program encourages students to engage in MVPA for at least 50% of PE.

The final CAP focused on the public school setting through Goose Creek Consolidated Independent School District (GCCISD). GCCISD is the leading public school system in Baytown and it is composed of 16 elementary, 5 junior high, and 6 high schools serving about 24,000 students. While GCCISD had previously implemented a coordinated school health approach, through Be Well Baytown, those efforts were amplified and delivered more consistently throughout the district. Additional training and resources were provided, new dedicated staff was hired, and an updated wellness policy was adopted. The school districts’ efforts were bolstered by multi-component, community-wide initiatives. While focused and measured most directly with the students in the schools, working within the district in tandem with multi-component interventions, a larger audience was reached including the families of the school children and the staff within the school district, which is the largest employer in the Baytown community.

### Evaluation plan

An evaluation plan, developed in partnership with RTI International, was created to ensure adequate data collection and align organizational objectives with outcomes [36]. Evaluation activities occurred at three levels: program impact, collective impact, and community impact, as outlined below.

### Statistical methods

#### Program impact

Program impact assessed the implementation of the evidence-based interventions. Quantitative and qualitative data on progress were collected from collaborating organizations via quarterly reports. Each organization completed a template indicating the implementation status of each evidence-based intervention. They described these in terms of procedural challenges, successes, and recommendations for sustainability. These reports also included information on the total reach, or the extent to which each intervention attracted its intended audience [37, 38]. Specifically, ‘reach’ included all people impacted directly by an evidence-based intervention in the CAP (e.g., students in the school district, adults served at food fairs, students at Lee College), divided by the total number of residents in Baytown, TX (75,992). The backbone team reviewed the reports quarterly to monitor progress and met with the collaborating organizations at least weekly to discuss progress and issues.

#### Collective impact

Success of the Collective Impact implementation assessed relational processes, shared goals, and facilitators for successful operations and management of the partnership. Data were collected by tracking attendance in the Steering Committee meetings and using an annual Steering Committee survey developed and administered by RTI International that measured: meeting shared goals, building new and sustaining existing partnerships, and identifying barriers and facilitators of implementation. Participants rated their confidence in the initiative meeting its shared goals on a scale of 1 (strongly disagree) to 5 (strongly agree), their perspectives on partnerships on a scale of 1 (strongly disagree) to 5 (strongly agree), and selected key barriers and facilitators from a pre-determined list.

#### Community impact

Community impact assessed the population-level behavioral impact. Considering GCCISD as a specific targeted community within Baytown, the district’s implementation of the CATCH program was assessed at baseline to understand K-8th grade students’ changes in diet and physical activity.

In year 1, four elementary schools in GCCISD piloted the full CATCH program (pilot schools) and all 21 elementary and junior high schools participated in the CATCH PE program. In year 2, the full CATCH program was expanded to include all 21 schools (expansion schools). The School Physical Activity and Nutrition (SPAN) Survey [39] was used to assess CATCH implementation. The System for Observing Fitness Instruction Time (SOFIT) [40] was used to assess CATCH PE. In year 1, the SPAN survey was administered to fourth and fifth grade students in the pilot schools. In year 2, the SPAN survey was administered to the fourth through sixth graders in the expansion schools and to fifth grade students at the pilot schools. SOFIT observations were conducted to assess changes in MVPA in a sample of seven PE classes in 2 elementary and 1 junior high schools before and after implementation. All assessment and analysis was conducted by the CATCH Global Foundation. Descriptive statistics were captured for student characteristics. The two-tailed student’s *t*-test were used to compare the tested parameters for SPAN survey results and activity levels for SOFIT, from baseline to follow-up. *P* values less than 0.05 were considered significant.

This project was approved by MD Anderson’s Quality Improvement Assessment Board*.* Informed consent was obtained when appropriate.

## Results

With support and strategic guidance from the Be Well Baytown Steering Committee, the collaborating organizations delivered 16 evidence-based interventions which reached 83% of the community with information and programming about sun safety, healthy living, and Be Well Baytown. Those reached through these interventions included individuals such as the 24,000 students and staff in GCCISD, the 7,500 students, faculty and staff at Lee College, the more than 14,000 individuals who received access to healthy food, and the thousands of individuals who had access to new walking trails, parks with sun shades, and educational programming offered at a variety of sites throughout the community. At the program, collective and community level these interventions resulted in positive change.

### Program impact

Based on partner reports, Be Well Baytown successfully implemented all 16 evidence-based interventions outlined in the CAP during the first 2 years (Table 2). This included 11 interventions focused on healthy eating and active living and 5 interventions focus on reducing UVR exposure. Half of the interventions (8) were delivered through GCCISD. By the conclusion of year 2, GCCISD completed their pilot and expanded CATCH, developed and executed a district-wide sun safety policy, and delivered sun safety programming for all elementary and junior high schools in the district, impacting more than 27,000 staff and students as well as their families.

The other 8 evidence-based interventions were aimed at the whole community. The YMCA provided community fitness programs and community-based social support for physical activity by providing walking clubs and the Diabetes Prevention Program. The United Way of Greater Baytown Area & Chambers County, working in partnership with a local food pantry, Hearts and Hands of Baytown, exceeded their annual goal for healthy food initiatives and delivered more than 1 million pounds of fresh produce through mobile food markets. As part of the multicomponent community-wide interventions to prevent skin cancer, Lee College educated their students and staff about the risks UVR exposure. Similarly, City of Baytown both educated their staff about the risks UVR exposure and provided outdoor workers with long-sleeved shirts and wide-brimmed hats to offer UVR protection.

Additionally, the physical environment was enhanced through improved infrastructure including the installation of four new walking trails at schools available to the community after-school hours, 20 sunscreen dispensers, and six sun shades at GCCISD schools, Lee College, and parks. Finally, three early child-care sites were selected to deliver nutrition and physical activity interventions by participating in the Outdoor Learning Environments! Texas Program to enhance their environments and educate their staff on healthy eating and outdoor learning. Further, these interventions created a strong foundation and momentum for working together to promote wellness in the community.

### Collective impact

Success of the Collective Impact of Be Well Baytown was assessed in two ways. First, attendance at the monthly Steering Committee meetings (11/2016 – 8/2019) was tracked. On average, 17 of the 25 members, or 68%, of the Steering Committee attended each meeting. Through these meetings, as well as additional interim dialogs, Steering Committee members fulfilled their responsibilities. At each meeting, collaborating organizations presented their work and Steering Committee members monitored and supported program implementation by suggesting ways to better connect the evidence-based interventions to the community. Further, Steering Committee members championed this work by sharing it on social media, in partners’ newsletters, and through their attendance at the annual, community-wide Be Well Baytown Day celebration.

Second, measurement of the implementation of the Collective Impact framework was tracked by using RTI International’s Steering Committee survey in August 2018, with 19 of 25 (76.0%) members participating. Results indicate that the Steering Committee is working positively toward achieving its shared goals. Most felt very (*n* = 13 [68.4%]) or completely (*n* = 4 [21.1%]) confident that the initiative would achieve its goal of “engaging the community in an ongoing dialog about the importance of healthy behaviors.” Most felt very (*n* = 10 [52.6%]) or completely (*n* = 5 [26.3%]) confident that the initiative would achieve its goal of “creating and advancing community-based strategies to improve cancer prevention and control.” Most felt very (*n* = 11 [57.9%]) or completely (*n* = 5 [26.3%]) confident that the initiative would achieve its goal of “increasing appropriate health behaviors and activities that can have a direct impact on cancer risk reduction.”

Survey participants of collaborating organizations (*n* = 12) answered questions about the initiative’s impact on partnerships. All participants somewhat or strongly agreed that they developed new partnerships (mean, 4.9/5), strengthened existing partnerships (mean, 4.9/5), and connected with individuals who will strengthen their organization’s work (mean, 4.9/5) through this initiative.

Furthermore, respondents ranked the three most and least helpful factors for implementation. The top-ranked facilitators were support from MD Anderson, having necessary resources and sufficient funding, and collaboration with other organizations. The most frequent barrier was “other” factors which included “red tape,” staff turnover, and time.

### Community impact

The community impact analysis focused on results from the SPAN assessment and the SOFIT observations within the population of GCCISD students. Demographics of the fourth, fifth, and sixth grade students who participated in the SPAN surveys are included in Table 3. Importantly, the results of the SPAN assessment indicated that CATCH implementation positively impacted several nutrition and physical activity outcomes in the 4 pilot and 17 expansion schools (Table 4). There was a notable increase in healthy food, vegetable, and water consumption as well as number of days of MVPA greater than 30 min.

**Table 3:** Demographics of the GCCISD students who completed the SPAN survey

	Pilot schools			Expansion schools	
	Year 1		Year 2		
	Sept 2017	May 2018	May 2019	Sept 2018	May 2019
Samples (n)	793	777	361	2,401	2,393
Grade					
Fourth	49.3%	49.3%	0%	45.3%	39.1%
Fifth	50.7%	50.7%	100.0%	43.7%	39.4%
Sixth	–	–	–	11.0%	21.5%
Sex					
Male	48.4%	47.5%	50.4%	49.9%	50.4%
Female	51.6%	52.5%	49.6%	50.1%	49.6%
Race/ethnicity					
White	9.7%	9.3%	7.8%	16.7%	17.4%
Black	16.4%	17.0%	19.4%	14.9%	14.4%
Hispanic	53.3%	56.7%	59.2%	43.9%	47.3%
Asian	0.4%	0%	0.3%	2.0%	2.3%
Native Hawaiian or other Pacific Islander	0.8%	0%	0%	0.4%	0.3%
American Indian or Alaskan Native	1.5%	1.0%	0%	1.3%	1.4%
Other	17.9%	15.5%	13.4%	20.8%	16.8%

**Table 4:** SPAN nutrition and physical activity results in GCCISD students

Variable	Mean number of times per day	
	Pilot schools (Sept 2017/May 2018/May 2019)	Expansion schools (Sept 2018/May 2019)
Nutrition outcomes		
Healthy food consumption	4.5/5.4*/5.1	4.2/4.6^a^
Unhealthy food consumption	5.4/4.8*/4.4	4.4/4.6
Fruit consumption	1.3/1.5^a^/1.4	1.2/1.3
Vegetable consumption	1.5/2.3^c^/2.0	1.5/1.8^b^
Drank water	1.6/1.7*/2.0^a^	1.7/1.9^b^
Physical activity outcomes		
Number of days of MVPA > 30 min	2.3/3.0^c^/3.4*	2.6/3.3^c^

*p < .05

^a^*p* < .01

^b^*p* < .001

^c^*p* < .0001

Results from the SOFIT observations indicated an increase in MVPA. Prior to implementation, students in these classes spent 45% of class time in MVPA. Post implementation, they engaged in MVPA for 51% of class time at the end of year 1, and 57% of class time after year 2. All classes reached the recommended threshold of 50% of class time in MVPA.

## Discussion

Over the first two years of Be Well Baytown, the evidence-based interventions implemented through the CAP had a positive impact at the program, collective, and community level.

At the program level, collaborating organizations successfully carried out evidence-based interventions and met their intended short-term outcomes increasing access to healthy food, providing education and programs to individuals of many ages, and improving the infrastructure to encourage physical activity and sun safety.

At the collective level, by working together to implement the CAP, the Steering Committee built a deep collaborative network and found new ways to work together. For example, prior to Be Well Baytown, mobile food markets were only held at one church. Subsequently, food markets were held at least bimonthly in parks and at schools and are supported by the Steering Committee. The Steering Committee volunteered at events, provided additional resources to attendees, and helped with recruitment for interventions that were supported by Be Well Baytown, such as soccer clubs, health promotional events at Lee College, educational programming. As one Steering Committee member stated, “This has been such an eye-opening experience to see firsthand how the collaborations seem to be multiplying and the result is better service, opportunity, and education for those we serve. These initiatives are helping to move families from a place of enablement to elevation. Turning the safety net into a trampoline!”

At the community level and for GCCISD students, a positive change is underway. Results for the more than 2,000 students who took the SPAN survey are consuming healthier foods, drinking more water, and being more physically active.

Be Well Baytown launched around the same time as Hurricane Harvey, a Category 4 hurricane that caused catastrophic flooding in Harris County. As a result, the implementation of many planned interventions was delayed. However, by end of the first 2 years, all interventions were deployed and evaluated. Additionally, this model is focused on interventions whose impact and outcomes are best measured over a longer-term, but this article reflects a short-term, single snapshot in time. In the future, additional community health metrics (e.g., obesity, sunburn frequency) will be assessed using multiple methods (e.g., city-level surveys, Health of Houston survey [41]). Additionally, while to date this work has been evaluated for its short-term effectiveness, in the future, an implementation science approach could be deployed to compare various methods for promoting the uptake of evidence-based interventions in community settings.

## Conclusions

Early success of Be Well Baytown indicates that leveraging the best practices of healthy community initiatives and applying them in a cancer prevention and control context can have a positive, short-term impact. This collaborative approach, led by the wants and needs of the community, has established an infrastructure that promises to improve long-term, cancer-related outcomes in this community. The general approach and components of this model could be leveraged by other cancer centers to improve their catchment area community’s health with a focus on cancer prevention. In Baytown, this work will continue to expand in scope to address other cancer risk factors (e.g., tobacco control, preventive care) and transition to the community to lead in sustaining the program’s interventions in 2025. MD Anderson will continue to refine and expand this model to mobilize communities to advance health and reduce cancer risks, with the goal to stop cancer before it starts.

## Data Availability

The data underlying this article will be shared on reasonable request to the corresponding author.
